# The Expression and Role Analysis of Methylation-Regulated Differentially Expressed Gene UBE2C in Pan-Cancer, Especially for HGSOC

**DOI:** 10.3390/cancers14133121

**Published:** 2022-06-25

**Authors:** Jiajia Li, Yating Sun, Xiuling Zhi, Qin Li, Liangqing Yao, Mo Chen

**Affiliations:** 1 Department of Gynecology Oncology, Obstetrics & Gynecology Hospital, Fudan University, Shanghai 200011, China; jiajia__li@163.com (J.L.); yating_s@163.com (Y.S.); liqin721003@163.com (Q.L.); 2 Department of Physiology and Pathophysiology, School of Basic Medical Sciences, Fudan University, Shanghai 200032, China; zhixiuling@fudan.edu.cn

**Keywords:** ovarian cancer, DNA methylation, UBE2C, bioinformatics

## Abstract

**Simple Summary:**

DNA methylation has attracted a great deal of scientific interest as an early biomarker and potential therapeutic target. HGSOC result in high mortality due to the absence of reliable biomarkers for early diagnosis and prognosis. In this study, we performed an integrated bioinformatic analysis and found that UBE2C was hypomethylation and overexpression in ovarian cancer, which was associated with advanced cancer stages and poor prognoses. Meantime, this finding was also confirmed in pan-cancer analysis. Furthermore, the experimental validation of the expression and role of UBE2C was performed on HGSOC tissues and cancer cell lines. Importantly, demethylation could upregulate the expression of UBE2C. Taken together, methylation-regulated UBE2C may be a novel biomarker for diagnosis and prognosis, not only for ovarian cancer but a variety of cancers.

**Abstract:**

High-grade serous ovarian cancer (HGSOC) is the most fatal gynecological malignant tumor. DNA methylation is associated with the occurrence and development of a variety of tumor types, including HGSOC. However, the signatures regarding DNA methylation changes for HGSOC diagnosis and prognosis are less explored. Here, we screened differentially methylated genes and differentially expressed genes in HGSOC through the GEO database. We identified that UBE2C was hypomethylation and overexpression in ovarian cancer, which was associated with more advanced cancer stages and poor prognoses. Additionally, the pan-cancer analysis showed that UBE2C was overexpressed and hypomethylation in almost all cancer types and was related to poor prognoses for various cancers. Next, we established a risk or prognosis model related to UBE2C methylation sites and screened out the three sites (cg03969725, cg02838589, and cg00242976). Furthermore, we experimentally validated the overexpression of UBE2C in HGSOC clinical samples and ovarian cell lines using quantitative real-time PCR, Western blot, and immunohistochemistry. Importantly, we discovered that ovarian cancer cell lines had lower DNA methylation levels of UBE2C than IOSE-80 cells (normal ovarian epithelial cell line) by bisulfite sequencing PCR. Consistently, treatment with 5-Azacytidine (a methylation inhibitor) was able to restore the expression of UBE2C. Taken together, our study may help us to understand the underlying molecular mechanism of UBE2C in pan-cancer tumorigenesis; it may be a useful biomarker for diagnosis, treatment, and monitoring, not only of ovarian cancer but a variety of cancers.

## 1. Introduction

High-grade serous ovarian cancer (HGSOC) is the most common ovarian cancer and is the leading cause of death from gynecologic cancers [1]. Most patients are diagnosed at the advanced stage and tend to develop a recurrence following standard clinical treatment in two years. The 5-year survival is < 40% and has not improved significantly over the last 30 years [2]. Furthermore, no novel biomarker has been approved for the screening, diagnosis, or monitoring of HGSOC in over two decades [3]. Therefore, a better understanding of HGSOC-specific molecular oncogenic mechanisms will provide information for improving the diagnosis and treatment of HGSOC.

It was proven that aberrant DNA methylation is a characteristic of various tumor types and serves as reliable earliest biomarkers in carcinogenesis [4,5]. As a common epigenetic modification mechanism in cancer, DNA methylation is a crucial regulator of gene transcription [6]. Aberrant DNA methylation alters gene expression and function, resulting in genome-wide abnormalities by interfering with the binding of transcription factors to the recognition position of gene promoters. It is generally believed that the hypermethylation of the tumor suppressor gene promoters inhibits corresponding gene expression and that hypomethylation of protooncogene promoter regions promotes corresponding gene expression. The DNA methylation of tumor suppressor genes and protooncogenes plays a role in the occurrence and progression of various tumor types [7,8]. These changes often occur before tumor formation or development, so identifying methylation-regulated differentially expressed genes (MeDEGs) based on high-throughput data has been considered to be of notable significance and can be considered biomarkers for the early diagnosis of tumors or predictors of high-risk cancer patients.

To our knowledge, limited studies have been performed regarding the cumulative analysis for MeDEGs of ovarian cancer using an array of data from multiple platforms. In the present study, we performed an analysis based on four gene expression profiling datasets (GSE69428, GSE18520, GSE54388, and GSE27651) and a gene methylation profiling dataset (GSE133556). We achieved 47 hub genes of MeDEGs by the Protein–Protein Interaction (PPI) network analysis and then performed the GO and KEGG analysis of these hub genes. We found that UBE2C was ranked not only top 13 in the hub genes but is also one of the most enriched pathways (cell cycle).

Ubiquitin-conjugating enzyme E2 C (UBE2C), as a member of the E2 ubiquitin-conjugating enzyme family, participates in the ubiquitination system, mitosis, and the regulation of the cell cycle by interacting with the anaphase-promoting complex/cyclostome (APC/C) [9]. A previous study from our group demonstrated that UBE2C was highly expressed in ovarian cancer tissues and promoted ovarian cancer progression by upregulating CDK1 [10]. Accumulating evidence has shown that UBE2C is highly expressed and acts as a proto-oncogene in various types of human cancers, such as breast cancer [11], lung cancer [12], esophageal cancer [13], colon cancer [14], and thyroid cancer [15]. However, the role of the UBE2C gene and the associated molecular mechanisms during tumorigenesis are unclear.

Here, we further investigate the expression of UBE2C, its relationship with prognosis, and DNA methylation in pan-cancers. Importantly, we want to verify the increased expression of UBE2C using clinical HGSOC clinical samples and ovarian cell lines. Additionally, we also tested the DNA methylation level of UBE2C in ovarian cell lines by bisulfite sequencing PCR and investigated the relationship between UBE2C expression and DNA methylation using DNA methylation inhibitor 5-Azacytidine. Furthermore, we obtained two UBE2C-related genes, CDC20 and MAD2L1, by determining the overlap between the sets of interacting proteins and coexpressed genes. As a result, the current data support an oncogenic function of UBE2C in pan-cancer tumorigenesis and may offer a new prognostic biomarker or therapeutic target for future development.

## 2. Materials and Methods

### 2.1. Microarray Data Information

The primary data from the DNA methylation profiling dataset GSE133556 (Infinium MethylationEPIC, based on GPL21145 platform) were downloaded from the Gene Expression Omnibus (https://www.ncbi.nlm.nih.gov/geo/; accessed 7 April 2020; GEO), which contained 99 HGSOC samples and 12 normal controls. The gene expression profiling datasets (GSE69428, GSE18520, GSE54388, and GSE27651; Affymetrix Human Genome U133 Plus 2.0 Array dataset, based on the GPL570 platform) were also acquired from the GEO database. The GSE69428 dataset included ten HGSOC samples and ten matched nontumor tissues; the GSE18520 dataset comprised 53 HGSOC samples and ten normal tissues; the GSE54388 dataset consisted of 16 HGSOC samples and six normal tissues; the GSE27651 dataset contained 22 HGSOC samples and six normal tissues.

### 2.2. Data Processing for Identification of DEGs, DMGs, and MeDEGs

First, the ChAMP package was used to read, filter, and standardize the original data from the GSE133556 methylation profile. During the filtering process, the following probes were sequentially removed: probes for non-CpG sites, all SNP-related probes, multihit probes, and X and Y chromosomes probes. Afterward, differentially methylated sites between the HGSOC group and the control group were selected according to the corrected *p*-value < 0.01. Subsequently, the DMGs were obtained by annotating the differentially methylated sites using the EPIC chip.

The differential expression between the HGSOC group and the control group from the chip expression data was analyzed using the limma package. Before the expression differences were examined, the probes were annotated; for cases where multiple probes corresponded to the same gene, the average value of multiple probes was taken as the expression level of the gene. The primary expression profile data were log2 transformed and normalized to obtain the Series Matrix File. The screening threshold for a significant difference in gene expression was a *p*-value < 0.05 and |log2Fold Change| > 0.585 (that is, a Fold Change >1.5 or a Fold Change < 1/1.5). Then, the genes that were significantly differentially expressed, either all upregulated or downregulated, in at least three expression datasets were selected as the final DEGs.

Finally, the intersection of the above-obtained hypermethylated genes and down-regulated genes and the overlap of hypomethylated genes and upregulated genes were examined to identify the MeDEGs.

### 2.3. Gene Ontology (GO) and Pathway Enrichment Analysis

Gene Ontology (GO) annotation and the Kyoto Encyclopedia of Genes and Genomes (KEGG) pathway analysis of all the MeDEGs and the hub genes were performed with the clusterprofile package. KEGG pathway analysis was performed to explore the involvement of MeDEGs or the hub genes in biological pathways. GO analysis was used to determine the relevant pathways in cellular components, biological processes, and specific molecular functions related to the MeDEGs or the hub genes. The pathview package was used to visualize the enrichment of MeDEGs on the KEGG pathways.

### 2.4. Protein-Protein Interaction (PPI) Network Construction and Module Analysis

The STRING (https://www.string-db.org/; accessed 20 June 2020) database was utilized to analyze the PPI of the MeDEGs at the highest confidence level of protein–protein interaction (combined score > 0.9). The hub genes in the PPI network, as well as the hub genes network, were identified by the Molecular Complex Detection (MCODE) plug-in and cytoHubba plug-ins.

### 2.5. Validation of the Screen Genes

Gene Expression Profiling Interactive Analysis (http://gepia2.cancer-pku.cn/; accessed 11 June 2021; GEPIA2.0) is an easy-to-use web tool that shows the gene expression based on the source of the Cancer Genome Atlas (TCGA) database. The single-gene expression level of the top 15 hub genes in ovarian cancer was examined via the GEPIA2.0. A fold change of >2 and a *p* value of < 0.01 were considered statistically significant.

Oncomine (http://oncomine.org/; accessed 11 June 2021) is a web-based data-mining platform that allows the analysis of differential gene expression for a majority of cancer types compared with respective normal tissues [16]. In the current study, the expression levels of the hub genes in ovarian cancer tumor tissues and normal tissues were verified using this platform.

### 2.6. Gene Expression Analysis

Using the Oncomine platform, the expression level of UBE2C in tumor tissues and healthy tissues was visualized. Then, UBE2C was evaluated with the “Gene_DE” module of the Tumor Immune Estimation Resource, version 2 website (http://timer.cistrome.org/; accessed 20 June 2021; TIMER2.0). The differences in the expression of UBE2C between the tumor tissues and the normal controls were analyzed for a variety of tumors and tumor subtypes from the TCGA. The GEPIA2.0 was utilized to generate box plots of the differences in expression between tumor tissues and the relevant normal tissues for the tumors without normal controls or highly limited normal tissues in TIMER2.0. A P value cutoff = 0.01, log2FC (Fold change) cutoff = 1, and “Match TCGA normal and GTEx data” were set. Violin plots of UBE2C expression were generated using the “Pathological Stage Plot” module of the GEPIA2.0 to show the relationship between UBE2C expression and the pathological stages (stage I, stage II, stage III, and stage IV) of all tumor types of TCGA database.

The Human Protein Atlas (https://www.proteinatlas.org/; accessed 15 June 2021; HPA) is a valuable tool for researchers studying protein localization and expression in human tissues and cells. We obtained the mRNA level of UBE2C in single-cell types with the “Cell type atlas” module. Meanwhile, we obtained the immunohistochemistry (IHC) staining of UBE2C in different types of tumor tissues and normal tissues of different tumor types by using the “Pathology Atlas” module and “Tissue Atlas” module, respectively. UBE2C expression in different tissues was quantitatively analyzed based on the degree of staining, staining intensity, and staining quantity provided by the HPA.

The UALCAN portal (http://ualcan.path.uab.edu/analysis-prot.html; accessed 23 June 2021) is a user-friendly web resource for analyzing cancer OMICS data, including the protein expression analysis based on the Clinical Proteomic Tumor Analysis Consortium (CPTAC) dataset [17]. In this study, we performed a protein expression analysis of UBE2C. All of the tumor types provided by the dataset were included. A *p*-value < 0.01 was regarded as statistically significant.

### 2.7. Survival Prognosis Analysis

The “Survival Map” module of the GEPIA2.0 was utilized to achieve the OS (overall survival) and RFS (disease-free survival) significance map data of UBE2C for all the tumor types of the TCGA database. The cut-off value for defining high and low was set at 50%, and the significant level was considered to be 0.05. The “survival analysis” module was also used to generate survival plots.

The Kaplan–Meier plotter (http://kmplot.com/analysis/; accessed 17 June 2021) was used to perform a survival analysis of the UBE2C expression for breast cancer, ovarian cancer, lung cancer, and gastric cancer. A *p*-value < 0.05 was regarded as statistically significant.

### 2.8. DNA Methylation Analysis of UBE2C

DNMIVD (at http://www.unimd.org/dnmivd/; accessed 21 June 2021) is a comprehensive annotation and interactive visualization database for DNA methylation profiles of diverse human cancers that draws on TCGA and GEO databases [18]. We searched for “UBE2C” in the “Quick Search” to obtain the methylation levels of the UBE2C promoter for specific cancers. The relationship between the methylation level and the expression level of UBE2C was displayed in the “Meth-Exp correlation” module. In the “survival” module, we performed the survival analysis based on the methylation level of UBE2C, using median DNA methylation beta values as a threshold to divide samples into high and low groups. The feature importance bar plot and the heatmap of the DNA methylation profile were generated in the diagnostic module.

### 2.9. UBE2C-Related Gene Enrichment Analysis

To obtain the top 100 similar genes correlated with UBE2C, we used the “Similar Genes Detection” module of the GEPIA2.0, choosing all the cancer types of the TCGA. The correlation analysis of the top 5 genes coexpressed with UBE2C was conducted via the “Correlation Analysis” of the GEPIA2.0, including all the cancer types from TCGA. A *p*-value < 0.01 was regarded as statistically significant. Subsequently, using the “Gene_Corr” module of “Exploration” in TIMER2.0, the correlations of UBE2C and the top 5 coexpressed genes were determined.

We searched the STRING website using “UBE2C” as a single protein and “Homo sapiens” as an organism. The proteins that can bind with UBE2C were obtained by setting the “Experiments” to active interaction sources, “low confidence (0.150)” as the minimum required interaction score, and “no more than 50 interactors” as the maximum number of interactors to display. The top 100 coexpressed genes from GEPIA2.0 and the 50 proteins that interacted with UBE2C from STRING were superimposed.

The Pathway Commons Network Visualizer (http://www.pathwacommons.org/pcviz/; accessed 26 June 2021; PCViz), a concise and efficient website, was used to perform the bioinformatics network analyses to describe the PPI. GO analysis and KEGG pathway analysis were conducted with OmicsBean (http://omicsbean.eicp.net:47349/; accessed 26 June 2021) software.

### 2.10. Quantitative Real-Time PCR (qRT-PCR)

The total RNA from ovarian cancer cell lines and tissues was extracted using Total RNA Extraction Reagent (Vazyme, Nanjing, China). The RNA was reverse-transcribed into complementary DNA using a ReverTra Ace qPCR RT Master Mix (TOYOBO, Shanghai, China). qRT-PCR was performed using SYBR Green Master Mix (Yeasen, Shanghai, China). GAPDH was used as an internal standard. The primers used in these studies were: UBE2C (forward: 5′ -GACCTGAGGTATAAGCTCTCGC- 3′, reverse: 5′- TTACCCTGGGTGTCCACGTT -3′) and GAPDH (forward: 5′- GGAGCGAGATCCCTCCAAAAT -3′, reverse: 5′-GGCTGTTGTCATACTTCTCATGG -3′).

### 2.11. Bisulfite Sequencing (BS) PCR

The next-generation sequencing-based BSP was performed to examine the gene DNA methylation following the previously reported method [19]. In brief, genomic DNA was extracted from ovarian cancer cells using the QIAamp DNA Mini Kit (QIAGEN, Hilden, Germany). Bisulfite sequencing (BS) was utilized to determine the DNA methylation status of the CpG islands of the promoter region of UBE2C. The bisulfite conversion of genomic DNA was performed using the ZYMO EZ DNA Methylation-Gold Kit (Zymo Research, Irvine, CA, USA) according to the manufacturer’s instructions. BSP primers of UBE2C were designed using the online MethPrimer software (http://www.urogene.org/methprimer/; accessed 25 June 2021) and listed in Table S1. Bisulfite-treated genomic DNA was used to amplify the CpG islands of the UBE2C promoter, which contains 47 CpG sites, using KAPA HiFi HotStart Uracil+ ReadyMix PCR Kit (Kapa Biosystems, Wilmington, MA, USA). Amplified PCR products were pooled in equal volumes, 5’-phosphorylated, 3’-dA-tailed, and ligated to a barcoded adapter using T4 DNA ligase (NEB). The barcoded libraries were sequenced on an Illumina platform.

### 2.12. Western Blot

The proteins in the tissues and cells were isolated using RIPA lysis buffer containing phenylmethylsulfonyl fluoride and protease inhibitor cocktail. The proteins were separated by SDS-PAGE and transferred to PVDF membranes before blocking with 5% milk. The membranes were incubated with primary antibody overnight at 4 °C and secondary antibody for 1 h at room temperature. Chemiluminescence was detected on a Tanon-5500 Imaging System (Tanon Science & Technology Ltd., Tanon, Shanghai, China). The primary antibodies used were rabbit polyclonal anti-UBE2C (ab252940, Abcam, Cambridge, MA, USA), mouse polyclonal anti-GAPDH (10494-1-AP, Proteintech, Wuhan, China), and rabbit polyclonal anti-tubulin (11224-1-AP, Proteintech, Wuhan, China).

### 2.13. RNA Interference

A2780 or SKOV3 cells were plated in six-well plates and transfected with five μM UBE2C siRNA (GenePharma, Shanghai, China) or control siRNA (GenePharma, Shanghai, China) using Lipofectamine 3000 (Invitrogen, Carlsbad, CA, USA). After 48 h, the mRNA and proteins in A2780 or SKOV3 cells were collected for validation. A knockdown efficiency above 80% was considered successful. The UBE2C-homo466: GGACCAUUCUGCUCUCCAUTT, AUGGAGAGCAGAAUGGUCCTT; UBE2C-homo505: CCAACA

UUGAUAGUCCCUUTT, AAGGGACUAUCAAUGUUGGTT; UBE2C-homo212: GUCU

GGCGAUAAAGGGAUUTT, AAUCCCUUUAUCGCCAGACTT.

### 2.14. Immunohistochemistry (IHC)

All samples, including 25 HGSOC tissues and 12 normal ovarian specimens, were obtained from the Obstetrics & Gynecology Hospital of Fudan University. Our study was approved by the institute’s Ethics Committee. The specimens were paraffin-embedded and sliced into 4 μm sections. The slides were heated for 1 h at 65 °C, deparaffinized in xylene for 40 min, and rehydrated through a series of graded ethanol solutions (100%, 95%, 80%, and 70%, each for 10 min). After incubation with 1% Triton and 3% hydrogen peroxide each for 10 min, Improved Antigen Retrieval Buffer (50 × Citrate Sodium Buffer, pH 6.0) (Yeasen, Shanghai, China) was used for antigen retrieval at 95 °C according to the instructions. After the slides were blocked with 5% donkey serum at room temperature for one hour, they were incubated with the primary antibody against human UBE2C (ab252940, Abcam, Cambridge, MA, USA) overnight at 4 °C. Following incubation with the secondary antibody at room temperature for one hour, the DAB Horseradish Peroxidase Color Development Kit (Beyotime Biotechnology, Shanghai, China) was used to detect the antibody binding according to the instructions before counterstaining with hematoxylin (Beyotime Biotechnology, Shanghai, China)and dehydration were performed. At least three fields, mainly containing cancer cells in each slide, were selected randomly for quantitative analysis. The score was calculated as the average staining intensity of all the selected cancer cells in each field. The evaluation of the protein expression was based on the staining score: (a) the percentage of positive cells in the tissue: 0 (0%), 1 (1–10%), 2 (11–50%), 3 (51–70%), or 4 (71–100%); (b) the staining intensity: 0 (none), 1 (weak), 2 (moderate), or 3 (strong). The staining score = (a) × (b).

### 2.15. Transwell Migration Assay

A total of 1 × 105 A2780 or SKOV3 cells were seeded in the upper chamber of a 24-well transwell chamber with an 8 μm pore polycarbonate membrane (Corning, Corning, NY, USA), and a double-serum medium was added to the lower chamber. After incubation for 24 h, the migrated cells on the lower chamber membrane surface were fixed with 4% paraformaldehyde (Solarbio, Beijing, China) for 10 min at room temperature. Then, the chamber was stained with 0.1% crystal violet (KeyGEN Biotech, Nanjing, China) for 30 min at room temperature. After the cells in the upper chamber were removed, images of the stained cells were acquired under an optical microscope. The migrated cells (crystal violet-stained cells) were counted in five random fields per well.

### 2.16. Statistical Analysis

All of the data were presented as mean ±  S.D. The Student’s *t*-test was used to evaluate the differences between the two groups. GraphPad Prism Software was used to analyze all data. A *p* < 0.05 was considered statistically significant.

## 3. Results

### 3.1. Identification of Methylation-Regulated Differentially Expressed Genes

**Figure 1:**
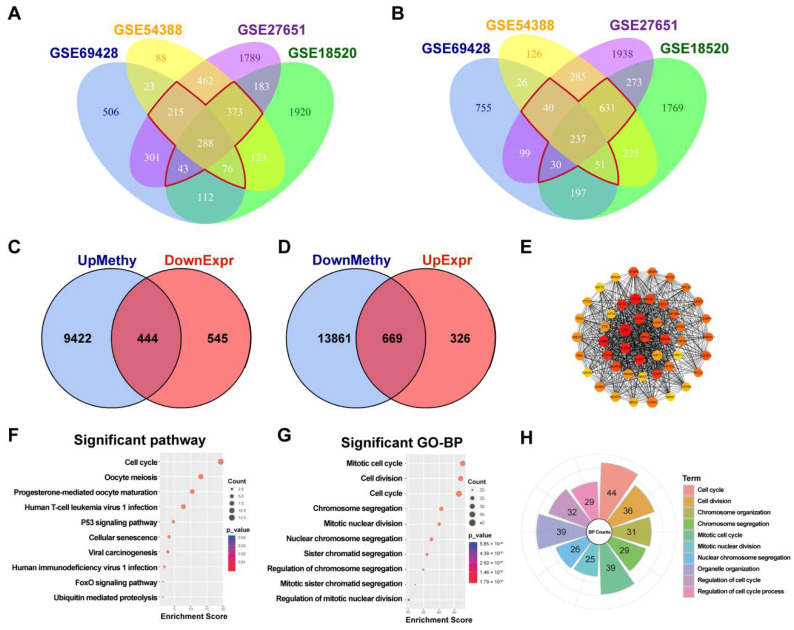
Identification of MeDEGs in HGSOC. (**A**). The intersection of the upregulated DEGs in the above four expression profiles; the red flower indicates the 995 genes that were upregulated in at least three of the expression profiling datasets. (**B**). The intersection of the downregulated DEGs in the above four expression profiles; the red flower indicates the 989 genes that were downregulated in at least three of the expression profiling datasets. (**C**). The overlap of hypermethylated DMGs and downregulated DEGs. (**D**). The overlap of hypomethylated DMGs and upregulated DEGs. (**E**). The top 47 hub genes with the highest connectivity, the color of the node indicates the connectivity in the PPI network, and the redder the color is, the greater the connectivity. (**F**). The KEGG analysis of the top 47 hub MeDEGs showed that the 47 hub genes were mainly enriched in the cell cycle pathway. (**G**,**H**). The GO analysis of the top 47 hub MeDEGs in biological processes (BP) showed that the 47 hub genes were mainly enriched in the cell cycle pathway; a total of 44 genes were enriched in the cell cycle pathway, including UBE2C.

A total of 2999 DEGs in the GSE69428 dataset were obtained: 1564 genes were upregulated, and 1435 genes were downregulated (Figure S1B, Table S2). In the GSE18520 dataset, 3118 genes were upregulated, and 3413 genes were downregulated (Figure S1B, Table S3). Among the 3269 DEGs in the GSE54388 dataset, 1648 genes were upregulated, and 1621 genes were downregulated (Figure S1B, Table S4). In the GSE27651 dataset, 3654 genes were upregulated, and 3533 genes were downregulated (Figure S1B, Table S5). To obtain more credible DEGs, we examined the overlap of DEGs from the four datasets and selected the genes with the same upregulated or downregulated trend in at least three of the datasets; these were the DEGs used in the final analysis. A total of 995 (indicated by the red flower in Figure 1A) upregulated genes and 989 (indicated by the red flower in Figure 1B) downregulated genes were obtained.

Moreover, by annotating these differentially methylated probes, we obtained 9866 DMGs with high methylation sites and 14,530 DMGs with low methylation sites (Table S6). The heatmap of the top 60 differently methylated sites is displayed in Figure S1A.

Then, 444 MeDEGs were obtained by examining the intersection of the hypermethylated genes and the downregulated genes (Figure 1C, Table S7). Similarly, 669 MeDEGs were obtained by determining the overlap of the sets of hypomethylated genes and upregulated genes (Figure 1D, Table S7). After analyzing the interactions of these 1113 MeDEGs using the STRING database with the highest protein–protein interaction confidence (combined score threshold was set to 0.9), we completed the PPI network construction using the Cytoscape tool and selected the hub genes from the PPI network using the MCODE plug-in. Then, the top 47 hub genes with the highest connectivity were obtained and sorted by degree using the cytoHubba plug-in for analysis (connectivity ≥ 30) (Figure 1E). We found UBE2C was overexpression with lower DNA methylation in HGSOC, ranking top 13 of the 47 hub genes (Figure S2A). Subsequently, we verified the expression of the top 15 genes by GEPIA2.0 (Figure S2B) and Oncomine (Figure S2C) and found that the relative mRNA levels of these genes were markedly higher in ovarian cancer tissues than in normal control tissues.

To explore the biological function of the top 47 hub genes, GO and KEGG analyses were performed using the R package cluster profile. The hub genes were mainly enriched in essential biological processes, especially in the cell cycle pathway (Figure S2D, Figure 1F). The GO-BP (biological processes) analysis showed that these hub genes were mostly related to the cell cycle and cell division, and 44 hub genes, including UBE2C, were enriched in the cell cycle pathway (Figure 1G,H, Table S8). A previous study by our group found that UBE2C was highly expressed in ovarian cancer and that downregulation of UEB2C induced higher apoptosis by blocking the G2/M transition [10]. In view of the significant hypomethylation of UBE2C in HGSOC from GEO datasets, we theorized that the increased expression of UBE2C in ovarian cancer was probably caused by DNA hypomethylation. Therefore, we selected UBE2C for further analysis.

### 3.2. The Expression of UBE2C in the Pan-Cancer Cohort

**Figure 2:**
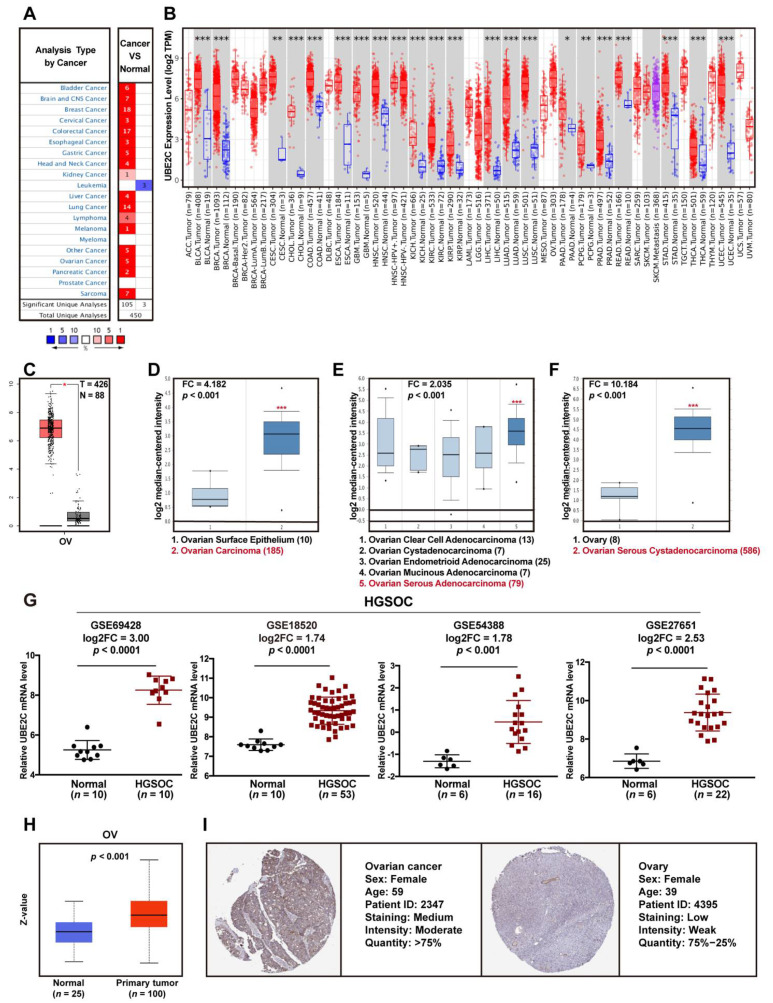
UBE2C gene and protein expression in ovarian cancer. (**A**). The mRNA expression of UBE2C was higher in almost all tumor types than in corresponding normal tissues, according to the analysis of the Oncomine database. (**B**). The mRNA levels of UBE2C were higher in all cancers than in normal tissues obtained from TIMER2.0. * *p* < 0.05; ** *p* < 0.01; *** *p* < 0.001 (Student’s *t*-test). (**C**). The mRNA expression of UBE2C in ovarian cancer by matching TCGA normal and GTEx data as controls in GEPIA2.0. * *p* < 0.01. (**D**). The mRNA levels of UBE2C were higher in ovarian carcinoma than in ovarian surface epithelium obtained from the Oncomine database (*p* < 0.001). (**E**). The mRNA expression of UBE2C was higher in ovarian serous adenocarcinoma than in other histopathological types of ovarian cancer derived from the Oncomine database (*p* < 0.001). (**F**). The mRNA expression of UBE2C was higher in ovarian serous cystadenocarcinoma than in ovarian tissue obtained from the Oncomine database (*p* < 0.001). (**G**). The mRNA expression of UBE2C in HGSOC and normal ovarian tissues from GSE69428, GSE18520, GSE54388, and GSE2765. (**H**). The protein levels of UBE2C were higher in ovarian cancer tissues than in normal tissues by CPTAC database analysis (*p* < 0.001). (**I**). IHC staining image of UBE2C in ovarian cancer and normal ovarian tissues obtained from the HPA database.

UBE2C was chosen for further pan-cancer analysis on the relationship between its expression in the TCGA cohort. As shown in Figure 2A, we noticed that ovarian cancer and the other 16 cancers had a significantly higher mRNA expression of UBE2C than normal tissues from Oncomine, except for leukemia. In all tumor types that had matched TCGA normal tissue data provided by the TIMER2.0 as controls, UBE2C was consistently upregulated in tumors compared to normal tissues (Figure 2B). This figure showed that ovarian cancer had the highest mRNA levels of UBE2C of almost all the cancers.

Since some cancer types lack TCGA normal controls in TIMER2.0, we used GEPIA2.0 to investigate UBE2C expression in these cancers by matching TCGA normal and GTEx data as controls. A significantly higher level of UBE2C mRNA was observed in ovarian cancer (OV; *p* < 0.01) (Figure 2C) and other cancer types, such as lymphoid neoplasm diffuse large B-cell lymphoma (DLBC; *p* < 0.01), acute myeloid leukemia (LAML; *p* < 0.01), thymoma (THYM; *p* < 0.01), skin cutaneous melanoma (SKCM; *p* < 0.01), advanced colorectal carcinoma (ACC; *p* < 0.01), uterine carcinosarcoma (UCS; *p* < 0.01), lower grade brain glioma (LGG; *p* < 0.01), testicular germ cell tumors (TGCT; *p* < 0.01), and sarcoma (SARC; *p* < 0.01), than in the corresponding normal tissues (Figure S3A). As shown in Figure 2D–F, UBE2C was highly expressed in ovarian carcinoma, especially in serous ovarian adenocarcinoma. In addition, in the above four gene expression profile datasets (GSE69428, GSE18520, GSE54388, and GSE27651), UBE2C was significantly overexpressed (Figure 2G).

To detect UBE2C protein expression levels in tumors, an analysis of the CPTAC database was performed with UALCAN. Compared to that in normal tissues, the level of UBE2C protein was obviously increased in tumor tissues of all cancer types available in the CPTAC database: OV (*p* < 0.001) (Figure 2H), BRAC (*p* < 0.001), colon cancer (*p* < 0.001), clear cell RCC (*p* < 0.001), uterine corpus endometrial carcinoma (UCEC; *p* < 0.001), and LUAD (*p* < 0.001) (Figure S3C). Moreover, the IHC staining of UBE2C in different forms of human cancer obtained from the HPA database also showed that UBE2C protein was upregulated in ovarian cancer (Figure 2I) and other tumor tissues compared to levels in the corresponding normal tissues (Figure S3B). In addition, we also found that UBE2C expression was correlated with tumor stage in KIRP (*p* < 0.001), KIRC (*p* < 0.001), KICH (*p* < 0.001), ACC (*p* < 0.001), TGCT (*p* < 0.05), LUAD (*p* < 0.01), LUSC (*p* < 0.05), and BRCA (*p* < 0.01) (Figure S4A).

### 3.3. Association of UBE2C Gene Expression with Prognosis in Ovarian Cancer

**Figure 3:**
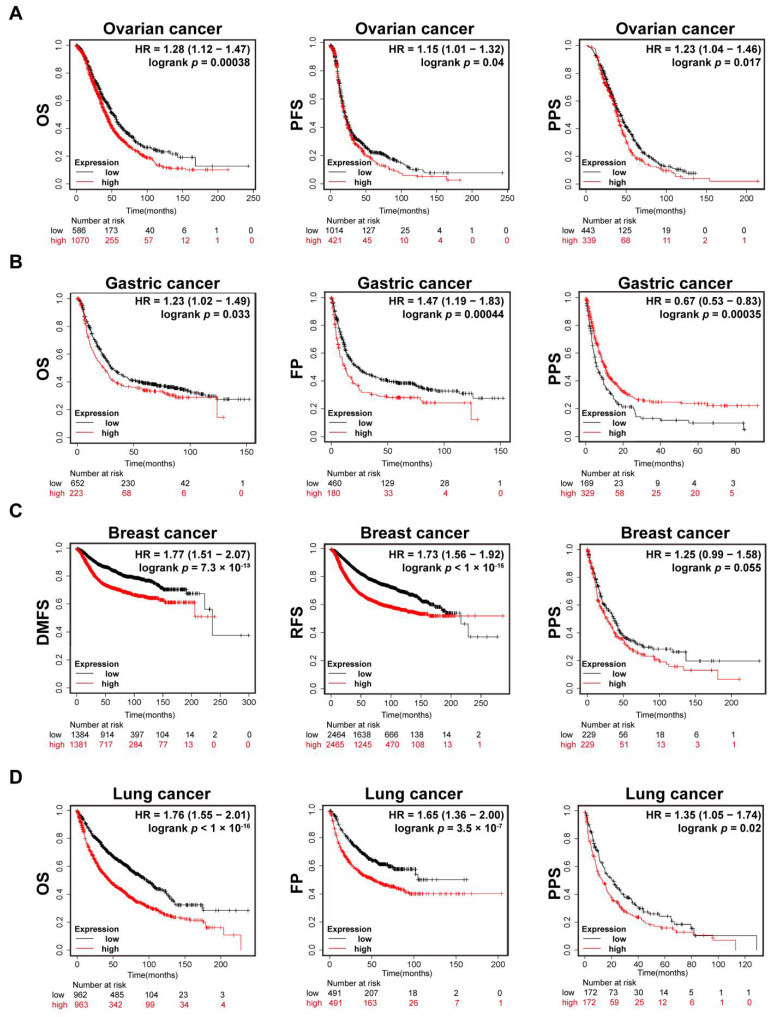
Prognosis analysis data of UBE2C in pan-cancer. (**A**). Kaplan–Meier plotter analysis of the relationship between UBE2C expression levels and clinical prognosis in ovarian cancer showed that higher UBE2C expression levels predicted poor overall survival (OS), progression-free survival (PFS), and post-progression survival (PPS). (**B**). Kaplan–Meier plotter analysis showed that higher levels of UBE2C were associated with worse OS and shorter times to first progression (FP) but better PPS in gastric cancer. (**C**). Kaplan–Meier plotter analysis showed that a higher level of UBE2C in breast cancer was related to worse distant metastasis-free survival (DMFS), disease-free survival (RFS), and PPS. (**D**). Kaplan–Meier plotter analysis suggested a strong relationship between the upregulation of UBE2C and poor OS, FP, and PPS in lung cancer.

The relationship between UBE2C expression and clinical survival was analyzed using TCGA datasets derived from the Kaplan–Meier plotter. As shown in Figure 3A, ovarian cancer patients with higher levels of UBE2C expression tended to have shorter OS (log-rank *p* = 0.00038), lower progression-free survival (PFS; log-rank *p* = 0.04), and worse post-progression survival (PPS; log-rank *p* = 0.0017). In gastric cancer, patients with higher UBE2C expression levels also had shorter OS (log-rank *p* = 0.033) and time to first progression(FP; log-rank *p* < 0.001) but better PPS(log-rank *p* < 0.001) (Figure 3B). A strong relationship between the upregulation of UBE2C and poor prognoses, such as RFS, distant metastasis-free survival (DMFS), PPS, OS, and FP, was also observed in BRCA and lung cancer (Figure 3C,D).

In ACC, BRCA, KIRC, KIRP, LGG, LUAD, MESO, PAAD, UVM, and SKCM, higher expression levels of UBE2C were related to worse OS (log-rank *p* < 0.05, Figure S4B). Additionally, disease-free survival (DFS; log-rank *p* ≤ 0.05, Figure S4C) was shorter for patients with higher UBE2C expression levels in ACC, BRCA, KIRC, KIRP, LGG, LIHC, MESO, PAAD, PRAD, THCA, UCEC, and UVM. The aforementioned results suggest that higher levels of UBE2C expression are associated with worse prognoses in various human cancers.

### 3.4. DNA Methylation Analysis of UBE2C

**Figure 4:**
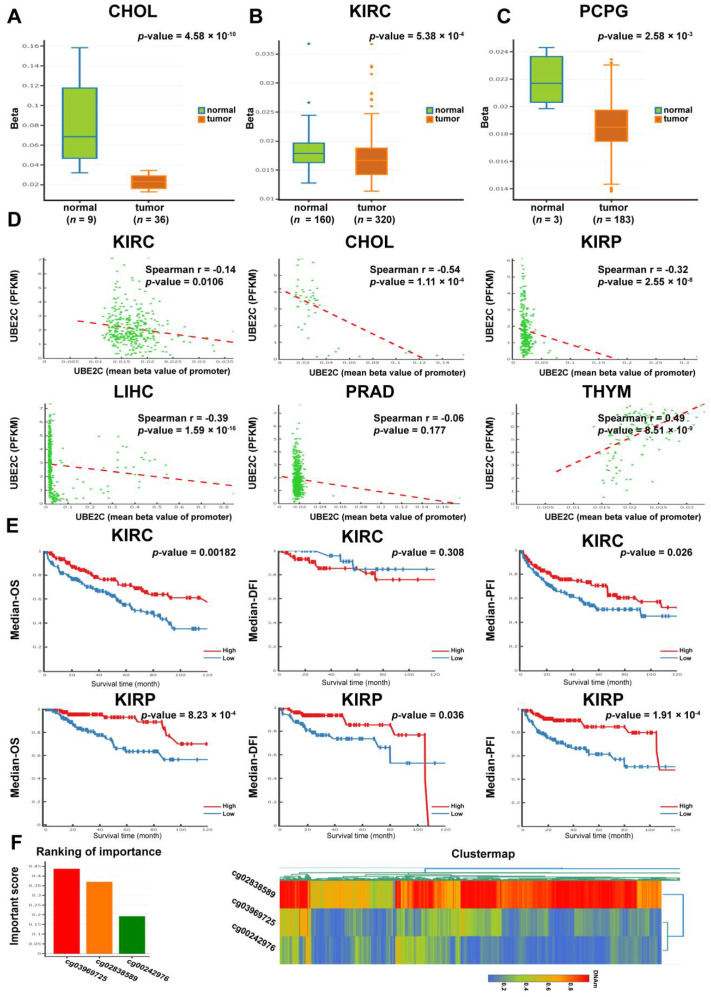
DNA methylation analysis data of UBE2C in pan-cancer. All analyses were performed by the DNMIVD website. (**A**–**C**). The DNA methylation levels of UBE2C in tumor tissues and normal tissues for CHOL (**A**), KIRC (**B**), and PCPG (**C**) (*p* < 0.01). (**D**). Spearman correlation analysis between the expression levels and DNA methylation levels of UBE2C in various cancer types (*p* < 0.05). (**E**). The association between the DNA methylation level of UBE2C and clinical prognosis (OS, DFI, and PFI) for KIRC and KIRP. (**F**). The top three UBE2C methylation sites (cg03969725, cg02838589, and cg00242976) with the highest importance scores (**left**). The DNA methylation cluster map (**right**) of these three sites showed that the cg02838589 site had the highest DNA methylation and the cg00242976 site had the lowest DNA methylation.

As UBE2C was hypomethylated in ovarian cancer, we investigated the methylation status of UBE2C in other cancer types. We first utilized the DNMIVD approach to analyze the difference in methylation of the UBE2C promoter region between tumor tissues and normal tissues for a variety of human cancers. The results demonstrated that the UBE2C promoter was hypomethylated in CHOL (Figure 4A), KIRC (Figure 4B), and PCPG (Figure 4C) tumor tissues compared to corresponding normal tissues (*p* < 0.01). In KIRC (*p* < 0.05), CHOL (*p* < 0.01), KIRP (*p* < 0.01), LIHC (*p* < 0.01), and PRAD (*p* = 0.117), the methylation levels were negatively correlated with the expression levels, while in THYM, the two were positively correlated (*p* < 0.01) (Figure 4D, Figure S5A). More importantly, the hypomethylation of UBE2C in KIRC and KIRP was correlated with poor survival, indicated by median OS, progression-free interval (PFI), and disease-free interval (DFI) (Figure 4E), although the median DFI in KIRC was not significantly different between the hypomethylation group and hypermethylation group (*p* = 0.308). Next, a logistic regression model was used to build a risk or prognosis model related to UBE2C methylation sites and screen a series of predictive methylation sites. The top three methylation sites were cg03969725 (Important Score = 0.44), cg02838589 (Important Score = 0.37), and cg00242976 (Important Score = 0.19) (Figure 4F).

### 3.5. Integral Analysis of UBE2C Related Genes

**Figure 5:**
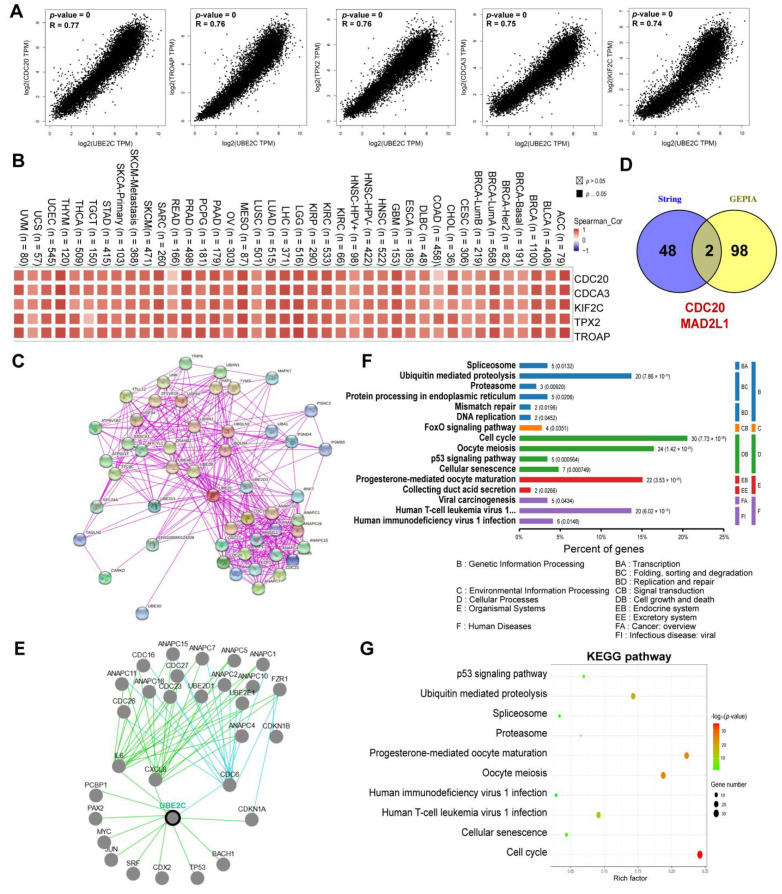
Coexpressed genes, targeted miRNA, and lncRNA of UBE2C (**A**). The correlation of UBE2C expression levels and CDC20, TROAP, TPX2, CDCA3, and KIF2C expression levels from the GEPIA2.0 website. *p* < 0.01. (**B**). Spearman correlation heatmap of the above five genes (CDC20, TROAP, TPX2, CDCA3, and KIF2C) and UBE2C for a variety of cancers. The results were obtained by TIMER2.0. (**C**). A total of 50 proteins that interact with UBE2C were obtained using STRING. (**D**). The intersection of the 50 proteins that interact with UBE2C and the 100 coexpressed genes identified two genes: CDC20 and MAD2L1. (**E**). The genes that controlled the expression (indicated by the green line) or phosphorylation (indicated by the blue line) of UBE2C were derived from the Pcviz pathway tool. (**F**,**G**). The GO analysis and KEGG analysis of the above 150 coexpressed or interacting genes by the OmicsBean commercial software showed that most of the enriched genes were in the cell cycle pathway.

To further explore the role of elevated UBE2C expression in pan-cancer tumorigenesis, we identified the top 100 genes coexpressed with UBE2C for all tumor types available in GEPIA2.0. The top five genes with the highest correlation with UBE2C were Cell division cycle 20 homolog (CDC20; R = 0.77), Trophinin-associated protein (TROAP; R = 0.76), Targeting protein for Xenopus kinesin-like protein 2 (TPX2; R = 0.76), Cell division cycle-associated protein-3 (CDCA3; R = 0.75), and Kinesin family member 2C (KIF2C; R = 0.74) (Figure 5A). The correlations of the top five genes with UBE2C in various cancer types are presented in Figure 5B. Following the screening of the 50 proteins that interact with UBE2C by the STRING tool (Figure 5C), we obtained an intersection of the top 100 coexpressed genes from GEPIA2.0 and the 50 proteins that interact with UBE2C from STRING. As shown in Figure 5D, CDC20 and mitotic arrest deficient 2-like 1 (MAD2L1) were identified. In addition, we also identified the genes that controlled expression (indicated by the green line) or phosphorylation (indicated by the blue line) with the Pcviz pathway tool (Figure 5E).

GO analysis and KEGG analysis were conducted on the abovementioned 150 coexpressed or interacting genes. From the results of the GO and KEGG analysis, it was determined that the molecular functions of these genes were mainly cellular processes, especially the regulation of the cell cycle, followed by oocyte meiosis, cellular senescence, and the p53 signaling pathway (Figure 5F,G). Additionally, genetic information processing, such as ubiquitin-mediated proteolysis and DNA replication, also enriched the molecular function of these genes (Figure 5F).

### 3.6. Verification of UBE2C Overexpression in HGSOC Samples

**Figure 6:**
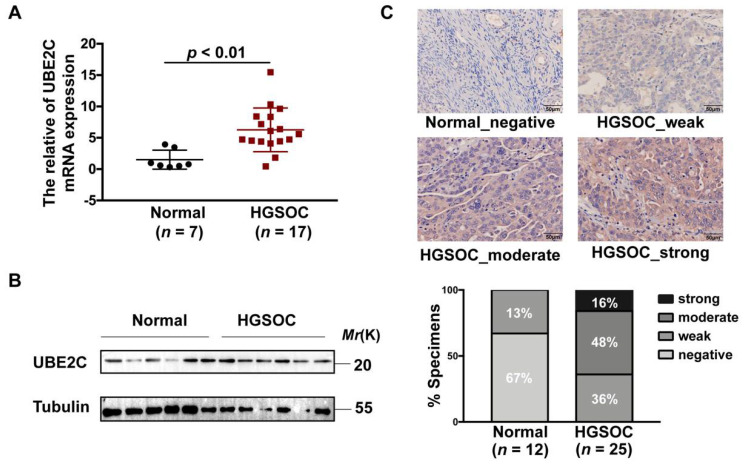
The upregulation of UBE2C in HGSOC tissues. (**A**). The mRNA expression levels of UBE2C were higher in clinical HGSOC samples (*n* = 17) than in normal ovarian tissues (*n* = 7) according to qRT–PCR analysis. The data are representative of three independent experiments. *p* < 0.01 vs. normal, *t*-test. (**B**). The protein levels of UBE2C were higher in clinical HGSOC samples (*n* = 5) than in normal ovarian tissues (*n* = 5) according to Western blot analysis (tubulin was used as the loading control). (**C**). Representative images (**above**) of UBE2C immunostaining in clinical HGSOC samples and normal ovarian tissues and the statistical chart of immunohistochemistry scores (**below**). The scale bar indicates 50 μm.

To further validate our results, we examined UBE2C mRNA and protein expression using normal ovarian tissues and HGSOC tissues obtained from the Obstetrics & Gynecology Hospital of Fudan University. We found that the average level of UBE2C mRNA in HGSOC tissues (*n* = 17) was significantly higher than that in normal ovarian tissues (*n* = 7) (Figure 6A). Consistent with these results, the protein levels of UBE2C were also elevated in HGSOC tissues (*n* = 5) compared with normal ovarian tissues (*n* = 5) (Figure 6B). The immunohistochemical analysis found that UBE2C mainly showed moderate and strong staining in HGSOC tissues (*n* = 25), while most of the normal tissues (*n* = 12) were not stained (Figure 6C).

### 3.7. The Promotion of UBE2C on Cell Migration and the Regulation of Hypomethylating Agents on UBE2C Expression in Ovarian Cancer

**Figure 7:**
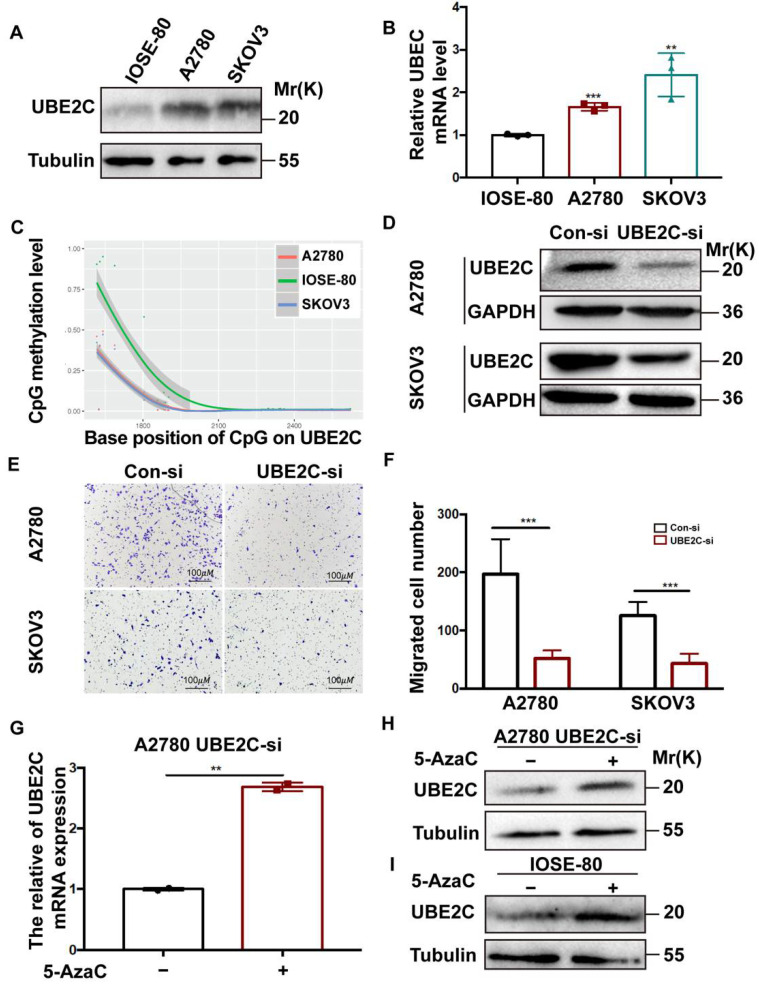
UBE2C promotes cell migration and hypomethylating agents regulate UBE2C expression. (**A**). Western blot analysis (tubulin was used as the loading control) of UBE2C expression in the normal ovarian epithelial cell line IOSE-80 and in ovarian cancer cell lines (A2780 and SKOV3). (**B**). The mRNA levels of UBE2C in IOSE-80, A2780, and SKOV3 cells according to qRT–PCR analysis. The data are representative of three independent experiments. ** p < 0.01, *** p < 0.001 vs. IOSE-80 cells; *t*-test. (**C**). The line chart of methylation levels of UBE2C for each sample (IOSE-80, A2780, and SKOV3) on each amplified fragment according to analysis of bisulfite sequencing PCR. (**D**). Western blot analysis (GAPDH was used as the loading control) of UBE2C expression in A2780 and SKOV3 cells following transfection with UBE2C-siRNA (UBE2C-si) or control-siRNA (Con-si). (**E**,**F**). Representative images (**E**) and the statistical chart (**F**) of migration of A2780 and SKOV3 cells transfected with UBE2C-si and Con-si. The data are representative of three independent experiments. *** p < 0.001 vs. Con-si; *t*-test. The scale bar indicates 100 μm. (**G**). The mRNA expression levels of UBE2C in A2780 cells transfected with UBE2C-si and treated with or without 5-AzaC (5 μM) for 72 h. DMSO was used as a vehicle. ** p < 0.01. (**H**). The protein expression levels of UBE2C in A2780 cells transfected with UBE2C-si treated with 5 μM 5-AzaC for 72 h. (**I)**. The protein expression levels of UBE2C in IOSE-80, which treated with 5 μM 5-AzaC for 72 h.

To verify the relationship between the expression levels and methylation levels of UBE2C in ovarian cancer, we first evaluated UBE2C expression levels in ovarian cancer cell lines (SKOV3 and A2780) and the normal ovarian epithelial cell line IOSE-80. Compared with the IOSE-80 cells, A2780 and SKOV3 cells had higher UBE2C mRNA and protein levels (Figure 7A,B). Then, to assess the DNA methylation levels of UBE2C in IOSE-80, A2780, and SKOV3 cells, we determined the methylation patterns of the CpG islands using BSP. The CpG islands in the promoter of UBE2C were predicted using online software and amplification products were confirmed by sequencing. Our results showed that IOSE-80 cells had the highest DNA methylation levels of UBE2C, and SKOV3 cells seemed to have the lowest methylation levels (Figure 7C, Table S9). We then silenced UBE2C expression in A2780 and SKOV3 cells (Figure 7D) and performed a Transwell cell migration assay to determine the effect of UBE2C on cell migration. As shown in Figure 7E,F, UBE2C knockdown led to a decrease in the migration ability of A2780 and SKOV3 cells. Additionally, the results of the previous bioinformatics analysis and bisulfite sequencing PCR clearly indicated the DNA hypomethylation status of UBE2C in ovarian cancer. To confirm that the expression levels of UBE2C were regulated by DNA methylation, we used 5-azacytidine (5-AzaC), a nucleoside analog of cytidine, to specifically inhibit DNA methylation. Due to the relatively low expression of UBE2C in existing cell lines (Figure 7A,B), A2780 cells were transfected with UBE2C siRNA and then treated with 5-AzaC (5 μM) for 72 h. As expected, 5-AzaC treatment significantly increased both the mRNA (Figure 7G) and protein expression levels (Figure 7H) of UBE2C in A2780 cells with UBE2C downregulation. In addition, we also conducted the above experiment in the IOSE-80 cells, which had the highest level of DNA methylation, and found that the expression of UBE2C was also increased after using the methylation inhibitor 5-AzaC (Figure 7I).

## 4. Discussion

Altered methylation has been reported to be an early and prevalent event in cancer development, and was widely recognized as an essential cancer-related biomarker and potential therapeutic target [20]. Therefore, the identification and analysis of methylation-regulated differentially expressed genes (MeDEGs) will be of great significance. Most previous studies have demonstrated that methylation in the promoter region tends to inhibit gene expression, while hypomethylation promotes gene expression [19,21]. Here, we identified 1113 MeDEGs by analyzing gene expression profiling datasets (GSE69428, GSE18520, GSE54388, and GSE27651) and gene methylation profiling dataset (GSE133556) of HGSOC tissues extracted from the GEO database. By analyzing the PPI network of these MeDEGs, we identified the hub genes, most of which were significantly upregulated and thus merit further study.

Among these 47 hub genes, ubiquitin-conjugating enzyme 2C (UBE2C) is a gene that we have previously reported. UBE2C was significantly upregulated in ovarian cancer, and downregulation of UBE2C inhibited cell proliferation, promoted cell apoptosis, induced G2/M cycle arrest, and reversed cisplatin resistance in vivo and in vitro experiments [10]. Meantime, overexpression of UBE2C has been demonstrated in various human malignancies, such as breast carcinoma, lung cancer, gastric cancer, etc [11,12,13,14]. Recently, there was an article on the bioinformatics analysis of UBE2C in pan-cancer [22]. Still, this article did not conduct a comprehensive and intensive analysis of UBE2C, only reporting the expression of UBE2C and its relationship with cancer stage and prognosis, as well as coexpressed genes in different cancers. The molecular mechanism underlying increased UBE2C gene expression in cancers is unclear and the relevant findings were not validated by experiments. Therefore, we conducted an integral analysis of the pan-cancer role of UBE2C with a variety of multibioinformatics tools, especially DNA methylation analysis. In accordance with previous studies, a global analysis of UBE2C expression found that upregulation of UBE2C was a common feature in human cancers and predicted invasive progression. Importantly, the overexpression of UBE2C was correlated with poor prognosis in a wide array of human cancers. Consistent with the above results, we verified that UBE2C was highly expressed in clinical HGSOC specimens and ovarian cell lines with Western blot analysis, qRT–PCR, and IHC. In addition, we found that downregulation of UBE2C could reduce ovarian cancer cell migration.

UBE2C plays a key role in the regulation of the cell cycle by partnering with APC/C to degrade mitotic cyclins. Surprisingly, our data confirmed that the genes that interacted or were coexpressed with UBE2C were mainly enriched in the regulation of the cell cycle, as well as oocyte maturation and meiosis, ubiquitin-mediated proteolysis, and DNA replication. This was consistent with our prior report showing cell cycle arrest in ovarian cancer cells by UBE2C downregulation [10].

Furthermore, by determining the overlap between the sets of interacting proteins and genes coexpressed with UBE2C, we identified two molecules: CDC20 and MAD2L1. Notably, as an activator of APC/C during mitosis, CDC20 is an important regulator of the cell cycle and plays a role in cancer emergence and development [23]. Consistent with the literature, CDC20 was coexpressed with UBE2C in human clear cell renal cell carcinoma [24]. MAD2L1 is a vital component of the spindle assembly checkpoint and tends to be overexpressed in many cancer types [25,26,27]. It can therefore be assumed that the two molecules might be able to explain the involvement of UBE2C with cancer progression and invasion.

The relationship between DNA hypomethylation and the mRNA overexpression of UBE2C, as well as the prognostic value of UBE2C hypomethylation, have previously been reported in a genome-wide study of CpG methylation in breast cancer samples [28]. Another genome-wide DNA methylation analysis in nonalcoholic steatohepatitis-related hepatocellular carcinomas found both hypomethylation of UBE2C promoters and UBE2C upregulation in hepatocellular carcinomas [29]. Recently, bioinformatics analyses have also discovered a UBE2C overexpression-hypomethylation correlation in hepatocellular carcinoma [30,31]. In agreement with the previous studies, our data found significant DNA hypomethylation of UBE2C in CHOL, KIRC, and PCPG tumor tissues and a negative correlation between the methylation levels of UBE2C and its expression levels in multiple cancer types. In addition, the hypermethylation level of UBE2C in cancers was associated with better follow-up survival in KIRC and KIPR. Importantly, our cell experiments also showed that the inhibition of DNA methylation upregulated the expression of UBE2C in ovarian cancer. Therefore, these results supported the potential functional effects of DNA hypomethylation on UBE2C upregulation in tumors. Hypomethylated UBE2C might serve as a potential new pan-cancer biomarker for the prognosis and diagnosis of various types of cancers. Therefore, we established a risk or prognosis model related to UBE2C methylation sites and identified three sites according to their importance score (cg03969725, cg02838589, and cg00242976).

However, there are still some limitations to this study. First, the MeDEGs were obtained by the bioinformatics analysis of the HGSOC gene methylation profiling dataset and gene expression profiling datasets from the GEO database. Second, we only verified that inhibiting DNA methylation increases the gene expression of UBE2C in ovarian cancer, ignoring other cancer types. Thus, further studies are necessary to elucidate exactly how DNA methylation regulates UBE2C expression in cancers.

In conclusion, we conducted a series of analyses on four gene expression profiling datasets and a gene methylation profiling dataset for HGSOC tissues and identified differentially expressed genes regulated by methylation. According to the identification of hub genes from the PPI network, we selected UBE2C for further bioinformatics analysis. Our pan-cancer analysis of UBE2C was the first to identify that the expression of UBE2C was consistently higher in almost all cancer types and that it was associated with cancer stage, clinical prognosis, and DNA methylation. Next, we established a risk or prognosis model related to UBE2C methylation sites and screened out the three sites. Finally, we verified the expression and role of UBE2C in HGSOC tissues with clinical specimens and cell functional experiments. Importantly, inhibiting DNA methylation was able to restore the expression of UBE2C in ovarian cancer. This finding, while preliminary, may help us to understand the underlying molecular mechanism of UBE2C in pan-cancer tumorigenesis, and UBE2C might be a diagnostic biomarker and a therapeutic target across many cancers.

## Data Availability

The datasets used and/or analyzed during the present study are available from the corresponding author on reasonable request.

## Supplementary Materials

The following are available online at https://www.mdpi.com/article/10.3390/cancers14133121/s1, Figure S1: The DMGs and DEGs from the methylation and expression profile datasets, Figure S2: Confirmation and functional enrichment analysis of hub MeDEGs in HGSOC tissues, Figure S3: The expression levels of UBE2C in the pan-cancer analysis, Figure S4: Stage atlas and survival analysis of UBE2C in the pan-cancer analysis, Figure S5: The relationship between UBE2C methylation and prognoses, Table S1: The BSP primers of UBE2C, Table S2: The DEGs in the GSE69428 dataset, Table S3: The DEGs in the GSE18520 dataset, Table S4: The DEGs in the GSE54388 dataset, Table S5: The DEGs in the GSE27651 dataset, Table S6: DMGs with high methylation sites and low methylation sites in the GSE133556 dataset, Table S7: MeDEGs in the GSE133556 dataset, Table S8: GO analysis of the hub genes, Table S9: The DNA methylation levels of UBE2C in IOSE-80, A2780, and SKOV3 cells.
