# Supplementary material for: The Expression and Role Analysis of Methylation-Regulated Differentially Expressed Gene UBE2C in Pan-Cancer, Especially for HGSOC
Source: Cancers (Basel). 2022 Jun 25;14(13):3121. doi: 10.3390/cancers14133121 (PMC9264902; doi:10.3390/cancers14133121)
Supplement: Supplementary file 1 [file cancers-14-03121-s001.zip › cancers-1717224-supplementary-Figures.pdf]

S.1

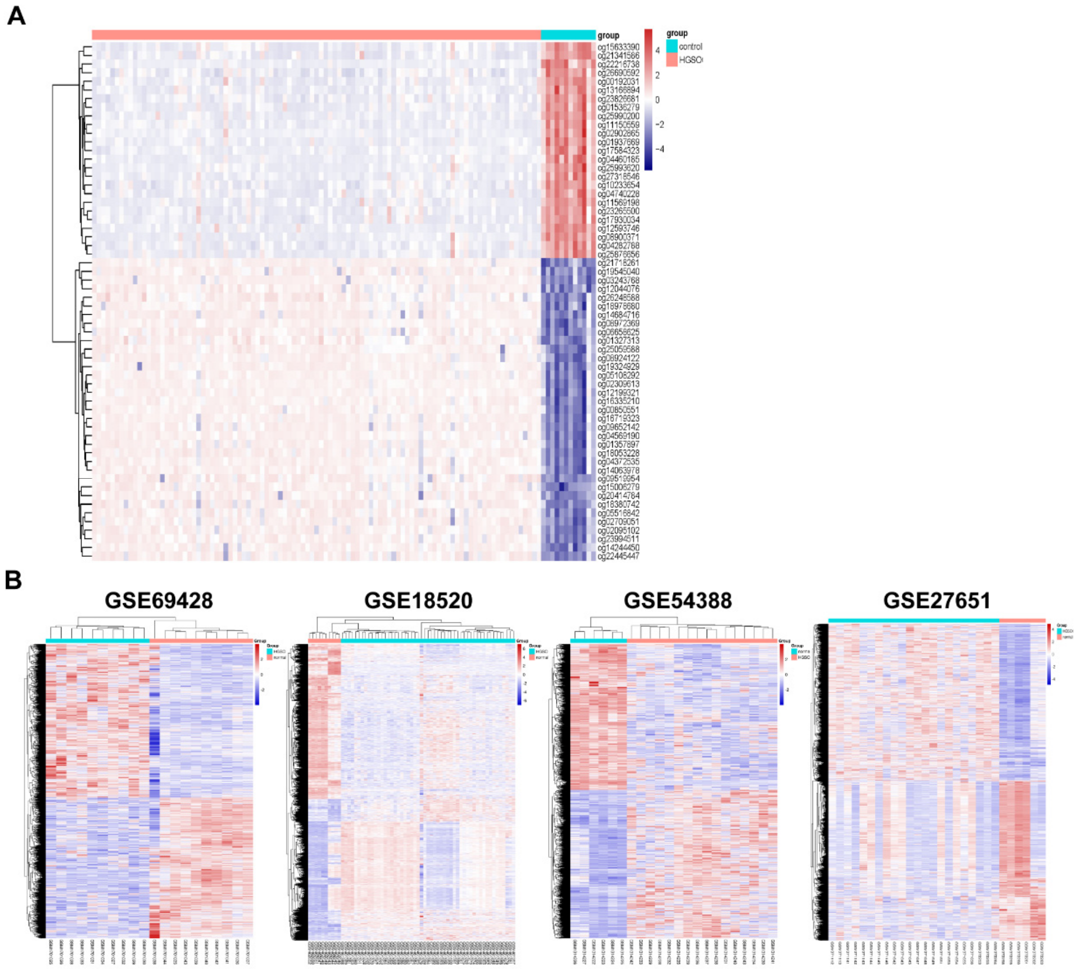


**Figure S1. The DMGs and DEGs from the methylation and expression profile datasets. A.** The heatmap of the top 60 differentially methylated sites between the HGSOC group and the control group of the GSE133556 dataset; the red color denotes hypermethylation, the blue color means hypomethylation. **B.** The heatmap of the DEGs between the HGSOC group and the control group of the GSE69428, GSE18520, GSE54388, and GSE27651 datasets.

**S.2**

**A**

| Node name | Degree |
|---|---|
| CDK1 | 95 |
| CDC20 | 81 |
| CCNA2 | 71 |
| CCNB1 | 70 |
| CCNB2 | 64 |
| BUB1B | 63 |
| BUB1 | 62 |
| AURKB | 61 |
| MAD2L1 | 59 |
| KIF11 | 57 |
| AURKA | 56 |
| NDC80 | 56 |
| UBE2C | 54 |
| KIF2C | 53 |
| CDCA8 | 52 |

**B**

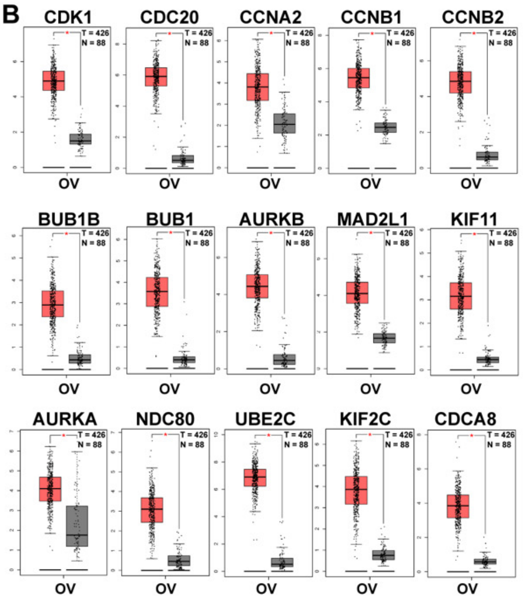


**C**

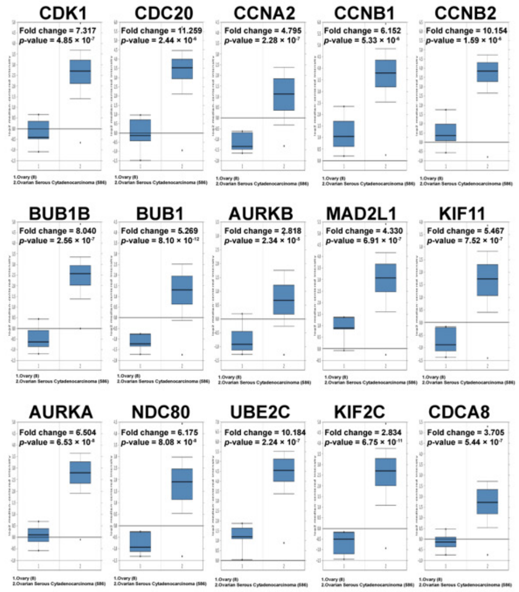


**D**

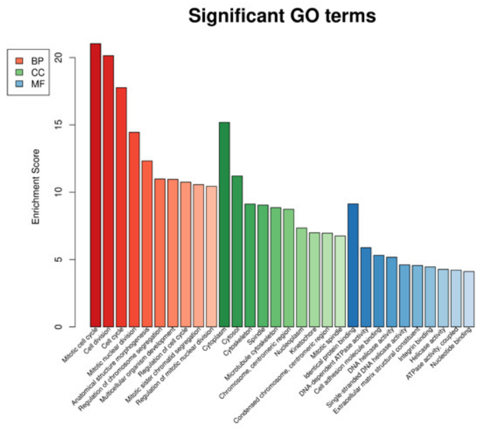


**Figure S2. Confirmation and functional enrichment analysis of hub MeDEGs in HGSOC tissues. A.** The top 15 hub MeDEGs sorted by degree. **B.** The expression analysis of the top 15 hub MeDEGs in ovarian cancer by the GEPIA2.0; the red color indicates the tumor tissues, and the gray color indicates the normal tissues; * $p < 0.01$. **C.** The top 15 MeDEGs expression analysis in ovarian serous cystadenocarcinoma and normal tissue via the Oncomine. **D.** The GO analysis of the top 47 hub MeDEGs.

S.3

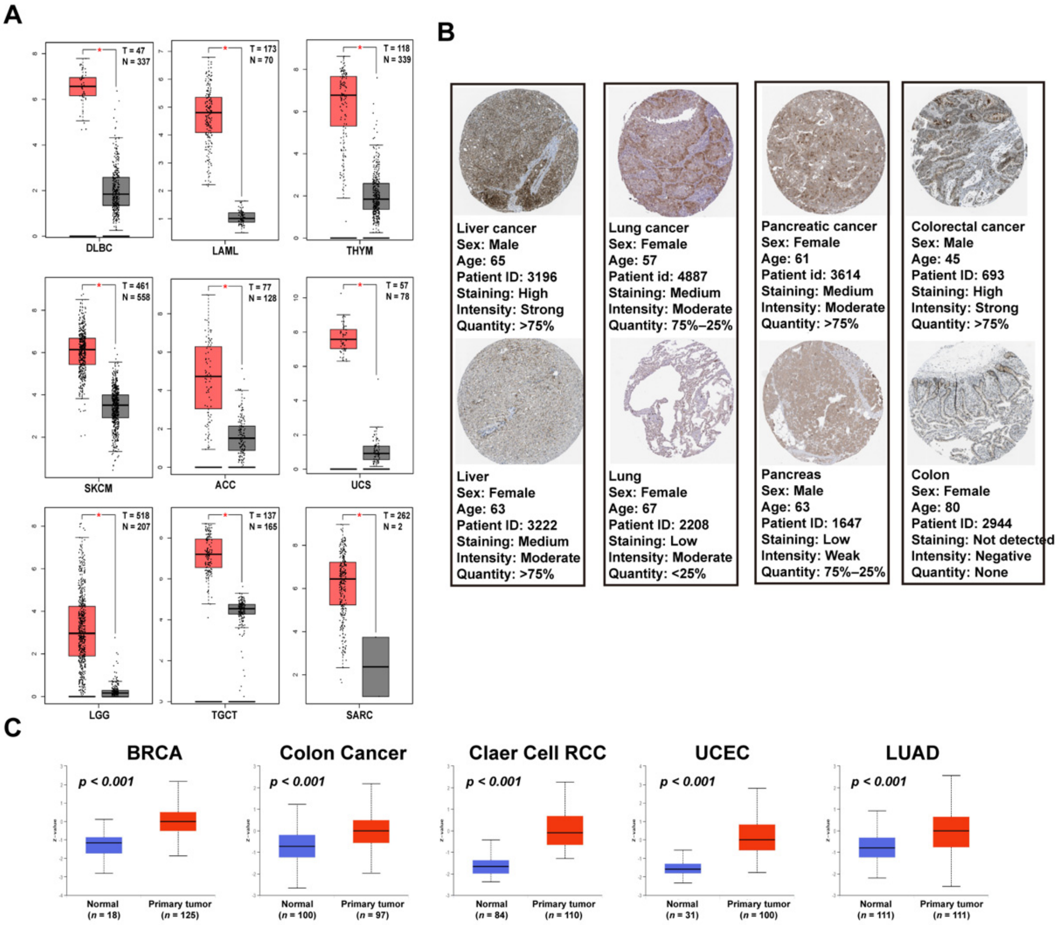


**Figure S3. The expression levels of UBE2C in the pan-cancer analysis. A.** The expression of UBE2C in various human cancers by matching TCGA normal and GTEx data as controls in GEPIA2.0. **p* < 0.01. B. The IHC staining image of UBE2C in tumor tissues and corresponding normal tissues of different human cancers obtained from the HPA database. C. The protein level of UBE2C in tumor tissues and normal tissues by CPTAC database analysis. *p* < 0.001

S.4

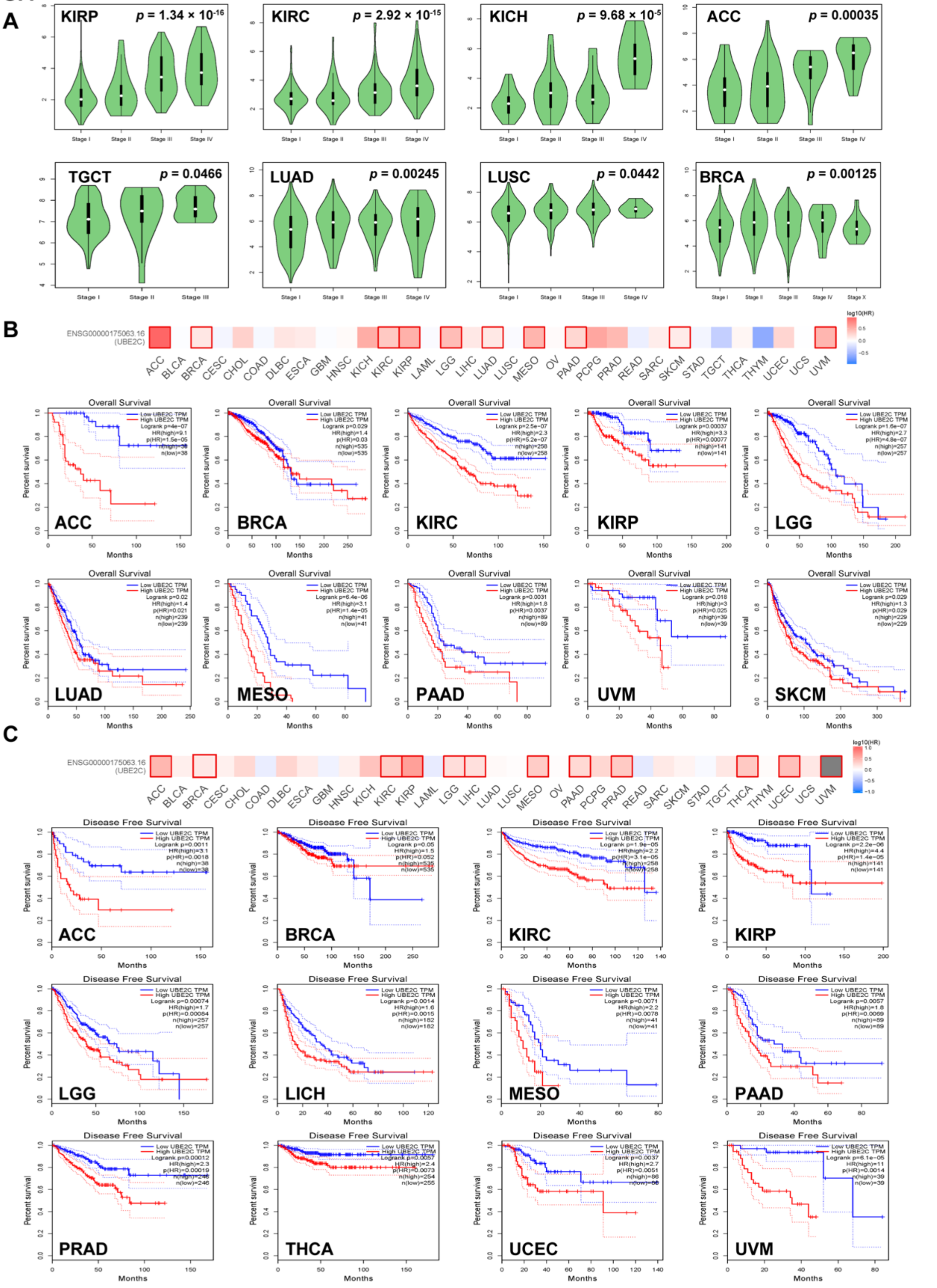


**Figure S4. Stage atlas and survival analysis of UBE2C in the pan-cancer analysis. A.** The correlation between UBE2C expression level and tumor stage by the GEPIA2.0 website. $p < 0.05$. **B-C.** The association between UBE2C expression and OS (**B**; log-rank $p < 0.05$) or DFS (**C**; log-rank $p \leq 0.05$) in different cancers by the GEPIA2.0.

**S.5**

**A**

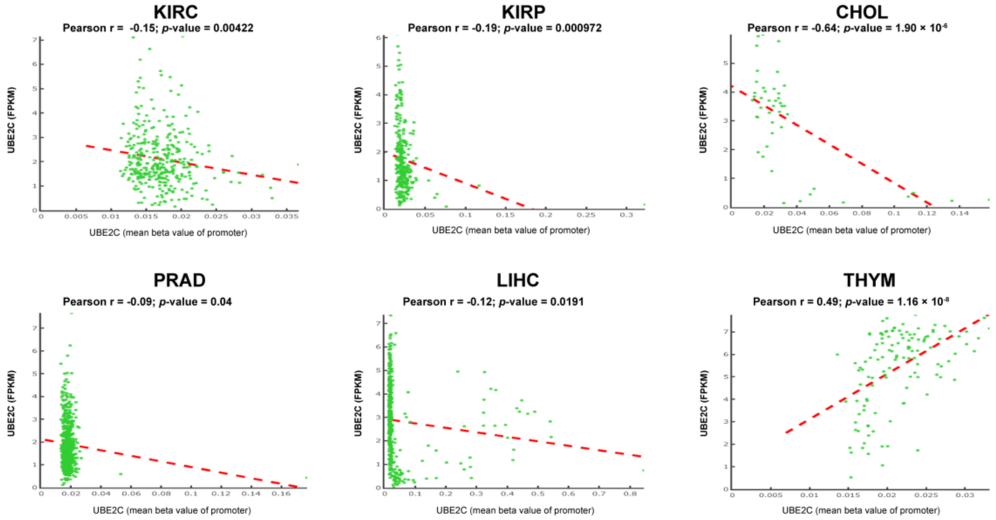


**Figure S5. The relationship between UBE2C methylation and prognoses. A.** The Pearson correlation between the expression level and DNA methylation level of UBE2C in KIRC (Kidney renal clear cell carcinoma), KIRP (Kidney renal papillary cell carcinoma), CHOL (Cholangiocarcinoma), PRAD (Prostate adenocarcinoma), LIHC (Liver hepatocellular carcinoma), and THYM (Thymoma).
